# Supplementary material for: A database of simulated tumor genomes towards accurate detection of somatic small variants in cancer
Source: PLoS One. 2018 Aug 30;13(8):e0202982. doi: 10.1371/journal.pone.0202982 (PMC6116990; doi:10.1371/journal.pone.0202982)
Supplement: S1 File — (DOCX) [file pone.0202982.s001.docx]

**Pre-tumor/normal sequencing data of NA12878**

To enhance analysis of different sequencing and subsequent mapping error profiles, we used four whole genomes and one exome Illumina data for the National Institute of Standards and Technology (NIST) reference material NA12878 as pre-tumors/normals. Two whole genomes (resulting BAM files are NA12878_HiSeq1_normal.bam and NA12878_HiSeq2_normal.bam) are part of a deep depth (~300x) dataset of 2x148 paired end reads, which is available at <ftp://ftp-trace.ncbi.nlm.nih.gov/giab/ftp/data/NA12878/NIST_NA12878_HG001_HiSeq_300x/> [1]. This dataset was from 14 libraries prepared from 6 vials of NA12878. The first and last tubes in the lot each generated 6 libraries: sample_U0a, sample_U0b, sample_U0c, sample_U5a, sample_U5b and sample_U5c. Two libraries each were made from four samples that were from each quarter of the lot at random: sample_U1a, sample_U1b, sample_U2a, sample_U2b, sample_U3a, sample_U3b, sample_U4a and sample_U4b. We used the folders [140115_D00360_0009_AH8962ADXX/](ftp://ftp-trace.ncbi.nlm.nih.gov/giab/ftp/data/NA12878/NIST_NA12878_HG001_HiSeq_300x/140115_D00360_0009_AH8962ADXX/), [140115_D00360_0010_BH894YADXX/](ftp://ftp-trace.ncbi.nlm.nih.gov/giab/ftp/data/NA12878/NIST_NA12878_HG001_HiSeq_300x/140115_D00360_0010_BH894YADXX/), [140127_D00360_0011_AHGV6ADXX/](ftp://ftp-trace.ncbi.nlm.nih.gov/giab/ftp/data/NA12878/NIST_NA12878_HG001_HiSeq_300x/140127_D00360_0011_AHGV6ADXX/) and [140127_D00360_0012_BH8GVUADXX/](ftp://ftp-trace.ncbi.nlm.nih.gov/giab/ftp/data/NA12878/NIST_NA12878_HG001_HiSeq_300x/140127_D00360_0012_BH8GVUADXX/) that contain sample_U1a, sample_U1b, sample_U2a and sample_U2b for NA12878_HiSeq1_normal.bam and sample_U3a, sample_U3b, sample_U4a and sample_U4b for NA12878_HiSeq2_normal.bam.

The exome data (resulting BAM file is NA12878_Exome_normal.bam) is of 2x100 paired ends and accessible from ftp://ftp-trace.ncbi.nlm.nih.gov/giab/ftp/data/NA12878/Garvan_NA12878_HG001_HiSeq_Exome/. It was from two libraries (NIST7035 and NIST7086) that were each sequenced on 2 lanes. The other two whole genomes (resulting BAM files are NA12878_ Illumina1_normal.bam and NA12878_ Illumina2_normal.bam) are from a high depth (more than 200x) dataset of 2x100 paired end reads [2]. The dataset was from the same library. Sequencing data of NA12878_ Illumina1_normal.bam were downloaded from the Platinum Genomes project in BaseSpace Sequence Hub. NA12878_ Illumina1_norma2.bam were from run accessions ERR174325, ERR174326, ERR174327, ERR174328 and ERR174330 at https://www.ebi.ac.uk/ena/data/view/ERS179577.

**Commands running BAMSurgeon**

**Running BAMSurgeon for generating addsnv and addindel input**

The desired genomic sites for SNV and indel spike-in were selected from each file with final target regions by running the python script randomsites.py in the BAMSurgeon distribution. The following are the commands for generating addsnv and addindel input:

Python ~/BAMSurgeon/scripts/randomsites.py –g ~/hg38.fa –b ~/final_target_regions.bed –n (the number of desired SNVs) –avoidN –s (a seed to make reproducible random picks) snv > ~/addsnv_input.bed

Python ~/BAMSurgeon/scripts/randomsites.py –g ~/hg38.fa –b ~/final_target_regions.bed –n (the number of desired indels) –avoidN –s (a seed to make reproducible random picks) indel > ~/addindel_input.bed

**Running BAMSurgeon for generating simulated tumors**

After generating addsnv and addindel input, we ran BAMSurgeon to introduce somatic SNVs and small indels to homozygous reference or wildtype sites in the pre-tumor/normal BAM file. The following are commands for generating a specific simulated tumor (NA12878_1_snv_indel_sorted.bam):

Python ~/BAMSurgeon/addsnv.py –r ~/hg38.fa –ignoresnps –tagreads –ignorepileup –picardjar ~/picard.jar –tmpdir ~/addsnv.tmp –aligner mem –minmutreads 1 –seed 1 –f ~/NA12878_HiSeq1_normal.bam –v ~/addsnv_input.bed –o ~/NA12878_1_snv.bam

Samtools sort –m 3G –T ~/temporarysnv -@ (the number of threads) –O BAM ~/NA12878_1_snv.bam > ~/NA12878_1_snv_sorted.bam

Samtools index ~/NA12878_1_snv_sorted.bam

Python ~/BAMSurgeon/addindel.py –r ~/hg38.fa –tagreads –ignorepileup –picardjar ~/picard.jar –tmpdir ~/addindel.tmp –aligner mem –minmutreads 1 –seed 1 –f ~/NA12878_1_snv_sorted.bed –v ~/addindel_input.bed –o ~/NA12878_1_snv_indel.bam

Samtools sort –m 3G –T ~/temporaryindel -@ (the number of threads) –O BAM ~/NA12878_1_snv_indel.bam > ~/NA12878_1_snv_indel_sorted.bam

Samtools index ~/NA12878_1_snv_indel_sorted.bam

**S1 Table.** Statistics of pre-tumor/normal BAM files.

| Pre-tumor/normal (BAM) | NA12878_HiSeq1_normal | NA12878_HiSeq2_normal | NA12878_Exome_normal | NA12878_ Illumina1_normal | NA12878_ Illumina2_normal |
| --- | --- | --- | --- | --- | --- |
| Number of reads | 1115089582 | 1058261027 | 165659753 | 1560021282 | 1561086461 |
| Secondary | 0 | 0 | 41681 | 0 | 0 |
| Supplementary | 0 | 0 | 0 | 0 | 0 |
| Mapped | 1106176130 (99.20%) | 1050129704 (99.23%) | 165602382 (99.97%) | 1554268164 (99.63%) | 1556112975 (99.68%) |
| Paired in sequencing | 1115089582 | 1058261027 | 165618072 | 1560021282 | 1561086461 |
| Properly paired | 1077605842 (96.64%) | 1020790018 (96.46%) | 164579224 (99.37%) | 1535399676 (98.42%) | 1538614610 (98.56%) |
| With itself and mate mapped | 1102102624 | 1045831896 | 165508374 | 1551610760 | 1554188936 |
| With mate mapped to a different chromosome | 16897562 | 17605912 | 126738 | 8762980 | 9326082 |

**S2 Table.** Distribution of the successful rate of somatic SNV and small indel spike-in for each simulated tumor.

| Simulated tumor | Successful rate | | Simulated tumor | Successful rate | |
| --- | --- | --- | --- | --- | --- |
|  | Somatic SNVs | Somatic small indels |  | Somatic SNVs | Somatic small indels |
| NA12878_1_snv_indel_sorted.bam | 0.936 | 1 | NA12878_2_snv_indel_sorted.bam | 0.932 | 0.995 |
| NA12878_3_snv_indel_sorted.bam | 0.939 | 0.995 | NA12878_4_snv_indel_sorted.bam | 0.935 | 0.997 |
| NA12878_5_snv_indel_sorted.bam | 0.939 | 0.997 | NA12878_6_snv_indel_sorted.bam | 0.937 | 0.997 |
| NA12878_7_snv_indel_sorted.bam | 0.938 | 0.993 | NA12878_8_snv_indel_sorted.bam | 0.936 | 0.997 |
| NA12878_9_snv_indel_sorted.bam | 0.933 | 1 | NA12878_10_snv_indel_sorted.bam | 0.934 | 0.993 |
| NA12878_11_snv_indel_sorted.bam | 0.934 | 0.993 | NA12878_12_snv_indel_sorted.bam | 0.936 | 0.995 |
| NA12878_13_snv_indel_sorted.bam | 0.937 | 0.994 | NA12878_14_snv_indel_sorted.bam | 0.935 | 0.996 |
| NA12878_15_snv_indel_sorted.bam | 0.936 | 0.996 | NA12878_16_snv_indel_sorted.bam | 0.937 | 0.997 |
| NA12878_17_snv_indel_sorted.bam | 0.939 | 0.994 | NA12878_18_snv_indel_sorted.bam | 0.941 | 0.994 |
| NA12878_19_snv_indel_sorted.bam | 0.935 | 0.994 | NA12878_20_snv_indel_sorted.bam | 0.935 | 0.993 |
| NA12878_21_snv_indel_sorted.bam | 0.935 | 0.987 | NA12878_22_snv_indel_sorted.bam | 0.937 | 0.989 |
| NA12878_23_snv_indel_sorted.bam | 0.937 | 0.993 | NA12878_24_snv_indel_sorted.bam | 0.936 | 0.994 |
| NA12878_25_snv_indel_sorted.bam | 0.939 | 0.994 | NA12878_26_snv_indel_sorted.bam | 0.936 | 0.996 |
| NA12878_27_snv_indel_sorted.bam | 0.937 | 0.995 | NA12878_28_snv_indel_sorted.bam | 0.934 | 0.997 |
| NA12878_29_snv_indel_sorted.bam | 0.934 | 1 | NA12878_30_snv_indel_sorted.bam | 0.932 | 1 |
| NA12878_31_snv_indel_sorted.bam | 0.938 | 0.991 | NA12878_32_snv_indel_sorted.bam | 0.936 | 1 |
| NA12878_33_snv_indel_sorted.bam | 0.939 | 0.991 | NA12878_34_snv_indel_sorted.bam | 0.939 | 0.991 |
| NA12878_35_snv_indel_sorted.bam | 0.928 | 0.995 | NA12878_36_snv_indel_sorted.bam | 0.932 | 0.995 |
| NA12878_37_snv_indel_sorted.bam | 0.935 | 0.996 | NA12878_38_snv_indel_sorted.bam | 0.933 | 0.994 |
| NA12878_39_snv_indel_sorted.bam | 0.933 | 0.991 | NA12878_40_snv_indel_sorted.bam | 0.929 | 0.996 |
| NA12878_41_snv_indel_sorted.bam | 0.934 | 0.996 | NA12878_42_snv_indel_sorted.bam | 0.933 | 0.995 |
| NA12878_43_snv_indel_sorted.bam | 0.931 | 0.994 | NA12878_44_snv_indel_sorted.bam | 0.935 | 0.995 |
| NA12878_45_snv_indel_sorted.bam | 0.939 | 0.994 | NA12878_46_snv_indel_sorted.bam | 0.931 | 0.996 |
| NA12878_47_snv_indel_sorted.bam | 0.932 | 0.998 | NA12878_48_snv_indel_sorted.bam | 0.933 | 0.995 |
| NA12878_49_snv_indel_sorted.bam | 0.936 | 0.996 | NA12878_50_snv_indel_sorted.bam | 0.935 | 0.995 |
| NA12878_51_snv_indel_sorted.bam | 0.937 | 0.996 | NA12878_52_snv_indel_sorted.bam | 0.935 | 0.994 |
| NA12878_53_snv_indel_sorted.bam | 0.938 | 0.996 | NA12878_54_snv_indel_sorted.bam | 0.937 | 0.994 |
| NA12878_55_snv_indel_sorted.bam | 0.954 | 0.944 | NA12878_56_snv_indel_sorted.bam | 0.954 | 0.889 |
| NA12878_57_snv_indel_sorted.bam | 0.925 | 1 | NA12878_58_snv_indel_sorted.bam | 0.948 | 1 |
| NA12878_59_snv_indel_sorted.bam | 0.959 | 0.944 | NA12878_60_snv_indel_sorted.bam | 0.948 | 0.944 |
| NA12878_61_snv_indel_sorted.bam | 0.942 | 1 | NA12878_62_snv_indel_sorted.bam | 0.942 | 0.944 |
| NA12878_63_snv_indel_sorted.bam | 0.948 | 0.944 | NA12878_64_snv_indel_sorted.bam | 0.952 | 0.954 |
| NA12878_65_snv_indel_sorted.bam | 0.929 | 0.954 | NA12878_66_snv_indel_sorted.bam | 0.940 | 0.954 |
| NA12878_67_snv_indel_sorted.bam | 0.931 | 0.977 | NA12878_68_snv_indel_sorted.bam | 0.942 | 0.954 |
| NA12878_69_snv_indel_sorted.bam | 0.959 | 0.977 | NA12878_70_snv_indel_sorted.bam | 0.929 | 1 |
| NA12878_71_snv_indel_sorted.bam | 0.954 | 0.954 | NA12878_72_snv_indel_sorted.bam | 0.956 | 1 |
| NA12878_73_snv_indel_sorted.bam | 0.946 | 0.965 | NA12878_74_snv_indel_sorted.bam | 0.948 | 0.977 |
| NA12878_75_snv_indel_sorted.bam | 0.945 | 0.954 | NA12878_76_snv_indel_sorted.bam | 0.943 | 0.954 |
| NA12878_77_snv_indel_sorted.bam | 0.943 | 0.977 | NA12878_78_snv_indel_sorted.bam | 0.948 | 0.954 |
| NA12878_79_snv_indel_sorted.bam | 0.945 | 0.931 | NA12878_80_snv_indel_sorted.bam | 0.944 | 0.954 |
| NA12878_81_snv_indel_sorted.bam | 0.926 | 0.919 | NA12878_82_snv_indel_sorted.bam | 0.894 | 0.986 |
| NA12878_83_snv_indel_sorted.bam | 0.888 | 0.989 | NA12878_84_snv_indel_sorted.bam | 0.889 | 0.991 |
| NA12878_85_snv_indel_sorted.bam | 0.906 | 0.980 | NA12878_86_snv_indel_sorted.bam | 0.904 | 0.982 |
| NA12878_87_snv_indel_sorted.bam | 0.898 | 0.996 | NA12878_88_snv_indel_sorted.bam | 0.900 | 0.993 |
| NA12878_89_snv_indel_sorted.bam | 0.896 | 0.993 | NA12878_90_snv_indel_sorted.bam | 0.905 | 0.984 |
| NA12878_91_snv_indel_sorted.bam | 0.893 | 0.990 | NA12878_92_snv_indel_sorted.bam | 0.898 | 0.988 |
| NA12878_93_snv_indel_sorted.bam | 0.898 | 0.986 | NA12878_94_snv_indel_sorted.bam | 0.892 | 0.982 |
| NA12878_95_snv_indel_sorted.bam | 0.895 | 0.985 | NA12878_96_snv_indel_sorted.bam | 0.890 | 0.986 |
| NA12878_97_snv_indel_sorted.bam | 0.900 | 0.988 | NA12878_98_snv_indel_sorted.bam | 0.897 | 0.989 |
| NA12878_99_snv_indel_sorted.bam | 0.896 | 0.986 | NA12878_100_snv_indel_sorted.bam | 0.895 | 0.988 |
| NA12878_101_snv_indel_sorted.bam | 0.893 | 0.988 | NA12878_102_snv_indel_sorted.bam | 0.896 | 0.986 |
| NA12878_103_snv_indel_sorted.bam | 0.898 | 0.986 | NA12878_104_snv_indel_sorted.bam | 0.897 | 0.987 |
| NA12878_105_snv_indel_sorted.bam | 0.897 | 0.990 | NA12878_106_snv_indel_sorted.bam | 0.898 | 0.991 |
| NA12878_107_snv_indel_sorted.bam | 0.899 | 0.992 | NA12878_108_snv_indel_sorted.bam | 0.896 | 0.986 |
| NA12878_109_snv_indel_sorted.bam | 0.892 | 0.983 | NA12878_110_snv_indel_sorted.bam | 0.896 | 0.980 |
| NA12878_111_snv_indel_sorted.bam | 0.886 | 0.971 | NA12878_112_snv_indel_sorted.bam | 0.901 | 0.984 |
| NA12878_113_snv_indel_sorted.bam | 0.899 | 0.978 | NA12878_114_snv_indel_sorted.bam | 0.901 | 0.993 |
| NA12878_115_snv_indel_sorted.bam | 0.903 | 0.985 | NA12878_116_snv_indel_sorted.bam | 0.899 | 0.986 |
| NA12878_117_snv_indel_sorted.bam | 0.899 | 0.982 | NA12878_118_snv_indel_sorted.bam | 0.898 | 0.983 |
| NA12878_119_snv_indel_sorted.bam | 0.898 | 0.982 | NA12878_120_snv_indel_sorted.bam | 0.895 | 0.986 |
| NA12878_121_snv_indel_sorted.bam | 0.895 | 0.986 | NA12878_122_snv_indel_sorted.bam | 0.895 | 0.988 |
| NA12878_123_snv_indel_sorted.bam | 0.903 | 0.985 | NA12878_124_snv_indel_sorted.bam | 0.902 | 0.983 |
| NA12878_125_snv_indel_sorted.bam | 0.899 | 0.990 | NA12878_126_snv_indel_sorted.bam | 0.897 | 0.982 |
| NA12878_127_snv_indel_sorted.bam | 0.896 | 0.983 | NA12878_128_snv_indel_sorted.bam | 0.895 | 0.985 |
| NA12878_129_snv_indel_sorted.bam | 0.894 | 0.986 | NA12878_130_snv_indel_sorted.bam | 0.896 | 0.987 |
| NA12878_131_snv_indel_sorted.bam | 0.898 | 0.986 | NA12878_132_snv_indel_sorted.bam | 0.901 | 0.983 |
| NA12878_133_snv_indel_sorted.bam | 0.898 | 0.987 | NA12878_134_snv_indel_sorted.bam | 0.899 | 0.986 |
| NA12878_135_snv_indel_sorted.bam | 0.899 | 0.979 |  |  |  |

References

1. Zook JM, Catoe D, McDaniel J, Vang L, Spies N, Sidow A, Weng Z, Liu Y, Mason CE, Alexander N et al: Extensive sequencing of seven human genomes to characterize benchmark reference materials. Sci Data 2016, 3:160025.

2. Eberle MA, Fritzilas E, Krusche P, Kallberg M, Moore BL, Bekritsky MA, Iqbal Z, Chuang HY, Humphray SJ, Halpern AL et al: A reference data set of 5.4 million phased human variants validated by genetic inheritance from sequencing a three-generation 17-member pedigree. Genome Res 2017, 27(1):157-164.
