# Supplementary material for: A database of simulated tumor genomes towards accurate detection of somatic small variants in cancer
Source: PLoS One. 2018 Aug 30;13(8):e0202982. doi: 10.1371/journal.pone.0202982 (PMC6116990; doi:10.1371/journal.pone.0202982)
Supplement: S1 Table — (DOCX) [file pone.0202982.s002.docx]

| Pre-tumor/normal (BAM) | NA12878_HiSeq1_normal | NA12878_HiSeq2_normal | NA12878_Exome_normal | NA12878_ Illumina1_normal | NA12878_ Illumina2_normal |
| --- | --- | --- | --- | --- | --- |
| Number of reads | 1115089582 | 1058261027 | 165659753 | 1560021282 | 1561086461 |
| Secondary | 0 | 0 | 41681 | 0 | 0 |
| Supplementary | 0 | 0 | 0 | 0 | 0 |
| Mapped | 1106176130 (99.20%) | 1050129704 (99.23%) | 165602382 (99.97%) | 1554268164 (99.63%) | 1556112975 (99.68%) |
| Paired in sequencing | 1115089582 | 1058261027 | 165618072 | 1560021282 | 1561086461 |
| Properly paired | 1077605842 (96.64%) | 1020790018 (96.46%) | 164579224 (99.37%) | 1535399676 (98.42%) | 1538614610 (98.56%) |
| With itself and mate mapped | 1102102624 | 1045831896 | 165508374 | 1551610760 | 1554188936 |
| With mate mapped to a different chromosome | 16897562 | 17605912 | 126738 | 8762980 | 9326082 |
