# Supplementary material for: A database of simulated tumor genomes towards accurate detection of somatic small variants in cancer
Source: PLoS One. 2018 Aug 30;13(8):e0202982. doi: 10.1371/journal.pone.0202982 (PMC6116990; doi:10.1371/journal.pone.0202982)
Supplement: S2 Table — (DOCX) [file pone.0202982.s003.docx]

| Simulated tumor | Successful rate | | Simulated tumor | Successful rate | |
| --- | --- | --- | --- | --- | --- |
|  | Somatic SNVs | Somatic small indels |  | Somatic SNVs | Somatic small indels |
| NA12878_1_snv_indel_sorted.bam | 0.936 | 1 | NA12878_2_snv_indel_sorted.bam | 0.932 | 0.995 |
| NA12878_3_snv_indel_sorted.bam | 0.939 | 0.995 | NA12878_4_snv_indel_sorted.bam | 0.935 | 0.997 |
| NA12878_5_snv_indel_sorted.bam | 0.939 | 0.997 | NA12878_6_snv_indel_sorted.bam | 0.937 | 0.997 |
| NA12878_7_snv_indel_sorted.bam | 0.938 | 0.993 | NA12878_8_snv_indel_sorted.bam | 0.936 | 0.997 |
| NA12878_9_snv_indel_sorted.bam | 0.933 | 1 | NA12878_10_snv_indel_sorted.bam | 0.934 | 0.993 |
| NA12878_11_snv_indel_sorted.bam | 0.934 | 0.993 | NA12878_12_snv_indel_sorted.bam | 0.936 | 0.995 |
| NA12878_13_snv_indel_sorted.bam | 0.937 | 0.994 | NA12878_14_snv_indel_sorted.bam | 0.935 | 0.996 |
| NA12878_15_snv_indel_sorted.bam | 0.936 | 0.996 | NA12878_16_snv_indel_sorted.bam | 0.937 | 0.997 |
| NA12878_17_snv_indel_sorted.bam | 0.939 | 0.994 | NA12878_18_snv_indel_sorted.bam | 0.941 | 0.994 |
| NA12878_19_snv_indel_sorted.bam | 0.935 | 0.994 | NA12878_20_snv_indel_sorted.bam | 0.935 | 0.993 |
| NA12878_21_snv_indel_sorted.bam | 0.935 | 0.987 | NA12878_22_snv_indel_sorted.bam | 0.937 | 0.989 |
| NA12878_23_snv_indel_sorted.bam | 0.937 | 0.993 | NA12878_24_snv_indel_sorted.bam | 0.936 | 0.994 |
| NA12878_25_snv_indel_sorted.bam | 0.939 | 0.994 | NA12878_26_snv_indel_sorted.bam | 0.936 | 0.996 |
| NA12878_27_snv_indel_sorted.bam | 0.937 | 0.995 | NA12878_28_snv_indel_sorted.bam | 0.934 | 0.997 |
| NA12878_29_snv_indel_sorted.bam | 0.934 | 1 | NA12878_30_snv_indel_sorted.bam | 0.932 | 1 |
| NA12878_31_snv_indel_sorted.bam | 0.938 | 0.991 | NA12878_32_snv_indel_sorted.bam | 0.936 | 1 |
| NA12878_33_snv_indel_sorted.bam | 0.939 | 0.991 | NA12878_34_snv_indel_sorted.bam | 0.939 | 0.991 |
| NA12878_35_snv_indel_sorted.bam | 0.928 | 0.995 | NA12878_36_snv_indel_sorted.bam | 0.932 | 0.995 |
| NA12878_37_snv_indel_sorted.bam | 0.935 | 0.996 | NA12878_38_snv_indel_sorted.bam | 0.933 | 0.994 |
| NA12878_39_snv_indel_sorted.bam | 0.933 | 0.991 | NA12878_40_snv_indel_sorted.bam | 0.929 | 0.996 |
| NA12878_41_snv_indel_sorted.bam | 0.934 | 0.996 | NA12878_42_snv_indel_sorted.bam | 0.933 | 0.995 |
| NA12878_43_snv_indel_sorted.bam | 0.931 | 0.994 | NA12878_44_snv_indel_sorted.bam | 0.935 | 0.995 |
| NA12878_45_snv_indel_sorted.bam | 0.939 | 0.994 | NA12878_46_snv_indel_sorted.bam | 0.931 | 0.996 |
| NA12878_47_snv_indel_sorted.bam | 0.932 | 0.998 | NA12878_48_snv_indel_sorted.bam | 0.933 | 0.995 |
| NA12878_49_snv_indel_sorted.bam | 0.936 | 0.996 | NA12878_50_snv_indel_sorted.bam | 0.935 | 0.995 |
| NA12878_51_snv_indel_sorted.bam | 0.937 | 0.996 | NA12878_52_snv_indel_sorted.bam | 0.935 | 0.994 |
| NA12878_53_snv_indel_sorted.bam | 0.938 | 0.996 | NA12878_54_snv_indel_sorted.bam | 0.937 | 0.994 |
| NA12878_55_snv_indel_sorted.bam | 0.954 | 0.944 | NA12878_56_snv_indel_sorted.bam | 0.954 | 0.889 |
| NA12878_57_snv_indel_sorted.bam | 0.925 | 1 | NA12878_58_snv_indel_sorted.bam | 0.948 | 1 |
| NA12878_59_snv_indel_sorted.bam | 0.959 | 0.944 | NA12878_60_snv_indel_sorted.bam | 0.948 | 0.944 |
| NA12878_61_snv_indel_sorted.bam | 0.942 | 1 | NA12878_62_snv_indel_sorted.bam | 0.942 | 0.944 |
| NA12878_63_snv_indel_sorted.bam | 0.948 | 0.944 | NA12878_64_snv_indel_sorted.bam | 0.952 | 0.954 |
| NA12878_65_snv_indel_sorted.bam | 0.929 | 0.954 | NA12878_66_snv_indel_sorted.bam | 0.940 | 0.954 |
| NA12878_67_snv_indel_sorted.bam | 0.931 | 0.977 | NA12878_68_snv_indel_sorted.bam | 0.942 | 0.954 |
| NA12878_69_snv_indel_sorted.bam | 0.959 | 0.977 | NA12878_70_snv_indel_sorted.bam | 0.929 | 1 |
| NA12878_71_snv_indel_sorted.bam | 0.954 | 0.954 | NA12878_72_snv_indel_sorted.bam | 0.956 | 1 |
| NA12878_73_snv_indel_sorted.bam | 0.946 | 0.965 | NA12878_74_snv_indel_sorted.bam | 0.948 | 0.977 |
| NA12878_75_snv_indel_sorted.bam | 0.945 | 0.954 | NA12878_76_snv_indel_sorted.bam | 0.943 | 0.954 |
| NA12878_77_snv_indel_sorted.bam | 0.943 | 0.977 | NA12878_78_snv_indel_sorted.bam | 0.948 | 0.954 |
| NA12878_79_snv_indel_sorted.bam | 0.945 | 0.931 | NA12878_80_snv_indel_sorted.bam | 0.944 | 0.954 |
| NA12878_81_snv_indel_sorted.bam | 0.926 | 0.919 | NA12878_82_snv_indel_sorted.bam | 0.894 | 0.986 |
| NA12878_83_snv_indel_sorted.bam | 0.888 | 0.989 | NA12878_84_snv_indel_sorted.bam | 0.889 | 0.991 |
| NA12878_85_snv_indel_sorted.bam | 0.906 | 0.980 | NA12878_86_snv_indel_sorted.bam | 0.904 | 0.982 |
| NA12878_87_snv_indel_sorted.bam | 0.898 | 0.996 | NA12878_88_snv_indel_sorted.bam | 0.900 | 0.993 |
| NA12878_89_snv_indel_sorted.bam | 0.896 | 0.993 | NA12878_90_snv_indel_sorted.bam | 0.905 | 0.984 |
| NA12878_91_snv_indel_sorted.bam | 0.893 | 0.990 | NA12878_92_snv_indel_sorted.bam | 0.898 | 0.988 |
| NA12878_93_snv_indel_sorted.bam | 0.898 | 0.986 | NA12878_94_snv_indel_sorted.bam | 0.892 | 0.982 |
| NA12878_95_snv_indel_sorted.bam | 0.895 | 0.985 | NA12878_96_snv_indel_sorted.bam | 0.890 | 0.986 |
| NA12878_97_snv_indel_sorted.bam | 0.900 | 0.988 | NA12878_98_snv_indel_sorted.bam | 0.897 | 0.989 |
| NA12878_99_snv_indel_sorted.bam | 0.896 | 0.986 | NA12878_100_snv_indel_sorted.bam | 0.895 | 0.988 |
| NA12878_101_snv_indel_sorted.bam | 0.893 | 0.988 | NA12878_102_snv_indel_sorted.bam | 0.896 | 0.986 |
| NA12878_103_snv_indel_sorted.bam | 0.898 | 0.986 | NA12878_104_snv_indel_sorted.bam | 0.897 | 0.987 |
| NA12878_105_snv_indel_sorted.bam | 0.897 | 0.990 | NA12878_106_snv_indel_sorted.bam | 0.898 | 0.991 |
| NA12878_107_snv_indel_sorted.bam | 0.899 | 0.992 | NA12878_108_snv_indel_sorted.bam | 0.896 | 0.986 |
| NA12878_109_snv_indel_sorted.bam | 0.892 | 0.983 | NA12878_110_snv_indel_sorted.bam | 0.896 | 0.980 |
| NA12878_111_snv_indel_sorted.bam | 0.886 | 0.971 | NA12878_112_snv_indel_sorted.bam | 0.901 | 0.984 |
| NA12878_113_snv_indel_sorted.bam | 0.899 | 0.978 | NA12878_114_snv_indel_sorted.bam | 0.901 | 0.993 |
| NA12878_115_snv_indel_sorted.bam | 0.903 | 0.985 | NA12878_116_snv_indel_sorted.bam | 0.899 | 0.986 |
| NA12878_117_snv_indel_sorted.bam | 0.899 | 0.982 | NA12878_118_snv_indel_sorted.bam | 0.898 | 0.983 |
| NA12878_119_snv_indel_sorted.bam | 0.898 | 0.982 | NA12878_120_snv_indel_sorted.bam | 0.895 | 0.986 |
| NA12878_121_snv_indel_sorted.bam | 0.895 | 0.986 | NA12878_122_snv_indel_sorted.bam | 0.895 | 0.988 |
| NA12878_123_snv_indel_sorted.bam | 0.903 | 0.985 | NA12878_124_snv_indel_sorted.bam | 0.902 | 0.983 |
| NA12878_125_snv_indel_sorted.bam | 0.899 | 0.990 | NA12878_126_snv_indel_sorted.bam | 0.897 | 0.982 |
| NA12878_127_snv_indel_sorted.bam | 0.896 | 0.983 | NA12878_128_snv_indel_sorted.bam | 0.895 | 0.985 |
| NA12878_129_snv_indel_sorted.bam | 0.894 | 0.986 | NA12878_130_snv_indel_sorted.bam | 0.896 | 0.987 |
| NA12878_131_snv_indel_sorted.bam | 0.898 | 0.986 | NA12878_132_snv_indel_sorted.bam | 0.901 | 0.983 |
| NA12878_133_snv_indel_sorted.bam | 0.898 | 0.987 | NA12878_134_snv_indel_sorted.bam | 0.899 | 0.986 |
| NA12878_135_snv_indel_sorted.bam | 0.899 | 0.979 |  |  |  |
